# Exploring the molecular causes of hepatitis B virus vaccination response: an approach with epigenomic and transcriptomic data

**DOI:** 10.1186/1755-8794-7-12

**Published:** 2014-03-11

**Authors:** Youtao Lu, Yi Cheng, Weili Yan, Christine Nardini

**Affiliations:** 1 Group of Clinical Genomic Networks, Key Laboratory of Computational Biology, CAS-MPG, Partner Institute for Computational Biology, Shanghai Institutes for Biological Sciences, Chinese Academy of Sciences, Shanghai, PR China; 2 Department of Clinical Epidemiology, Children’s Hospital of Fudan University, Shanghai, PR China

**Keywords:** Hepatitis B virus, Vaccine, Methylation, Omics

## Abstract

**Background:**

Variable responses to the Hepatitis B Virus (HBV) vaccine have recently been reported as strongly dependent on genetic causes. Yet, the details on such mechanisms of action are still unknown. In parallel, altered DNA methylation states have been uncovered as important contributors to a variety of health conditions. However, methodologies for the analysis of such high-throughput data (epigenomic), especially from the computational point of view, still lack of a gold standard, mostly due to the intrinsic statistical distribution of methylomic data i.e. binomial rather than (pseudo-) normal, which characterizes the better known transcriptomic data.

We present in this article our contribution to the challenge of epigenomic data analysis with application to the variable response to the Hepatitis B virus (HBV) vaccine and its most lethal degeneration: hepatocellular carcinoma (HCC).

**Methods:**

Twenty-five infants were recruited and classified as good and non-/low- responders according to serological test results. Whole genome DNA methylation states were profiled by Illumina HumanMethylation 450 K beadchips. Data were processed through quality and dispersion filtering and with differential methylation analysis based on a combination of average methylation differences and non-parametric statistical tests. Results were finally associated to already published transcriptomics and post-transcriptomics to gain further insight.

**Results:**

We highlight 2 relevant variations in poor-responders to HBV vaccination: the hypomethylation of RNF39 (Ring Finger Protein 39) and the complex biochemical alteration on SULF2 via hypermethylation, down-regulation and post-transcriptional control.

**Conclusions:**

Our approach appears to cope with the new challenges implied by methylomic data distribution to warrant a robust ranking of candidates. In particular, being RNF39 within the Major Histocompatibility Complex (MHC) class I region, its altered methylation state fits with an altered immune reaction compatible with poor responsiveness to vaccination. Additionally, despite SULF2 having been indicated as a potential target for HCC therapy, we can recommend that non-responders to HBV vaccine who develop HCC are quickly directed to other therapies, as SULF2 appears to be already under multiple molecular controls in such patients. Future research in this direction is warranted.

## Background

DNA methylation (addition of a methyl group to the 5th carbon cytosine residues in CpGs islands) is stably maintained, inheritable and regarded as an epigenetic marker, which augments stability and diversity of biological phenotypes, yet without modifying the genomic sequence. DNA methylation not only plays a crucial role in a spectrum of physiological processes, such as gene imprinting and X-chromosome inactivation [1], but is also associated with diseases including cancer, autoimmune maladies and psychiatric disorders [2].

Bisulfite-conversion based approaches are widely used for DNA methylation measurements, and exploit both microarray and sequencing technologies, as it is the case for other *omics.* Examples include Illumina HumanMethylation 450 K beadchip [3] for the former, and whole genome short-gun bisulfite sequencing (GWSBS [4]) and reduced representation bisulfite sequencing (RRBS [5]) for the latter, all offering fine resolution (down to the single nucleotide).

The degree of methylation is usually denoted as β, ranging from 0 to 1. Methylation data are presented in the same matricial form of expression data (locus × sample), however, cautions must be used in the direct application of transcriptiomic analysis tool to methylation data. In particular, the assumption that most genes are not differentially expressed no longer holds for methylation data: in the human genome 70% to 80% of CpGs are methylated to various extents [6]. Furthermore, the overall expression in a transcriptome is generally assumed to be invariant, which is the principle for ratio-intensity (R-I) plots [7], but this is not the case for methylation data where the total amount of CpG methylation may also differ substantially across individuals [6]. Most importantly, unlike gene expression data, which are generally assumed to be normally or log-normally distributed, DNA methylation data present a peculiar bimodal distribution, which breaks the normality assumption and defies the applications of Gaussian distribution based statistical approaches such as *t*-test or ANOVA. Although both SAM (Significance Analysis of Microarray, [8]) and LIMMA (Linear Models for Microarray Data, [9]) utilize moderated *t*-statistic and do not need the assumption of rigorous normality, their sensitivity is generally affected by a non-normal distribution.

Despite these difficulties, the ubiquity of methylation phenomena makes them interesting candidates to explain number of open clinical problems. In particular, Hepatitis B virus (HBV) vaccine is an effective prevention of HBV infection, yet not all people can benefit from it because of varying degrees of responsiveness. We have already shown [10] that genetic effects have a dominant role in such a response, however, the characterization of the phenomenon is far from being complete, and we here propose to enlarge the picture to epigenetic (methylation) aspects.

Given the importance of the clinical phenomenon (infection rate in Southeast Asia, parts of China and tropical Africa above >8% [11]) and the numerous computational issues involved in the analysis of methylation data, we chose to adopt a custom pipeline, based on multiple filtering and non-parametric statistics to rank differentially methylated (DM) loci in 25 infants showing different responses to the HBV vaccine. Further, as a mean to better filter the list of DMs, we backed this analysis with published transcriptional (mRNA screens) and post-transcriptional (miRNA screens) data to gain more insight into the molecular effects of altered methylation.

## Methods

### Ethical statement

The study protocols and consent procedure were approved by The Medical Ethical Committee of Children’s Hospital of Fudan University, Shanghai, China. Written informed consent forms were obtained from parents on the behalf of the participants involved in the study, conducted in accordance with the guidelines proposed in the World Medical Association Declaration of Helsinki.

### Whole-genome DNA methylation data & study subjects

All subjects were recruited at their 1-year-old regular physical examination performed at the children-care clinics of five comprehensive hospitals in Urumqi (Xinjiang Uygur Autonomous Region, China) after they received the 5 μg recombinant HBV vaccine recommended by the Chinese Ministry of Health (KT60, 2004 L00065, Kangtai Biological Product, Shenzhen, Guangdong, China). Sampling was done at each of the three injections, following the national 0-1-6 HBV vaccination schedule. The inclusion and exclusion of subjects, HBV biomarker examination and data collection are described elsewhere [10].

Twenty-five infants were selected for genome-wide DNA methylation analysis. Thirteen of these were non- or low-responders to the vaccine (cases, anti-HBs < 100mIU/ml) and 12 were normal responders (controls, anti_HBs > 500mIU/ml). Whole blood (2 ml) was collected for testing DNA methylation levels using the Illumina HumanMethylation 450 K microarray, processed and filtered according to the standard Illumina protocol in 2 batches: the first with 17 samples and the second with 8 samples. Clinical data are listed in Additional file 1 and expression data are deposited at the National Center for Biotechnology Information Gene Expression Omnibus (GEO, [12]) public repository http://www.ncbi.nlm.nih.gov/geo/query/acc.cgi?acc=GSE48300.

### Methylomic data preprocessing

**Figure 1:**
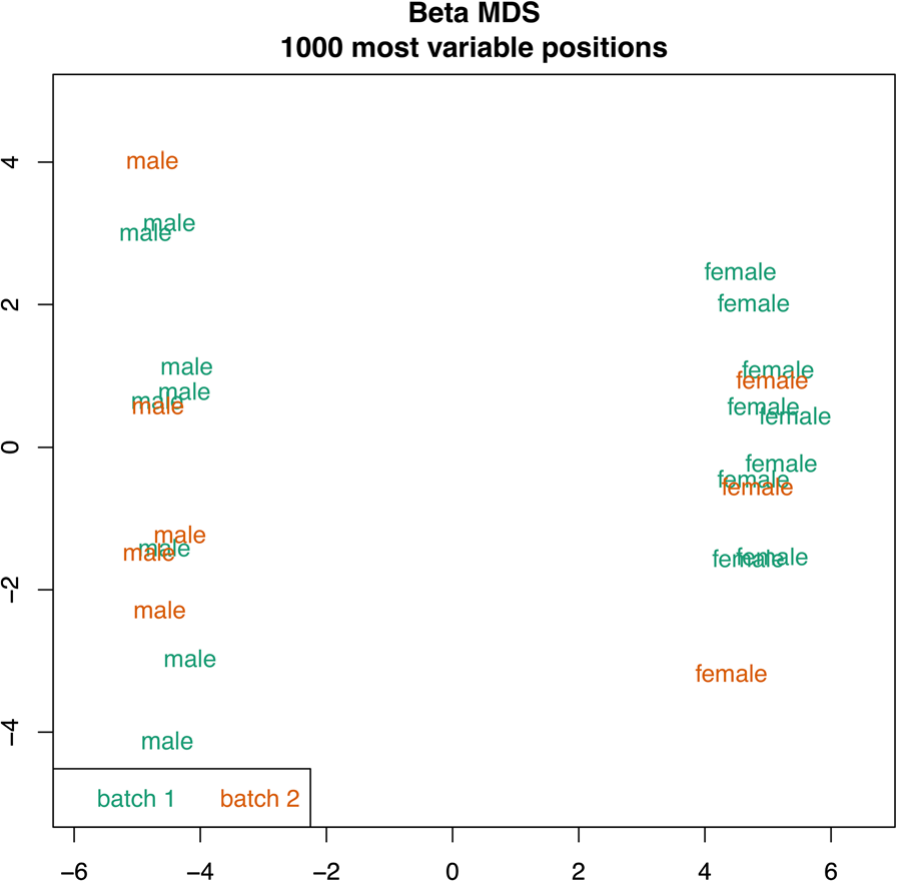
**MDS plot.** Multiple dimensional scaling (MDS) plot of the 1000 most variable loci showing that no significant batch effect can be detected, while samples genders are easily discriminated.

Quality Control (QC) assessment was performed with the open-source R package *minfi*[13]. Data distribution and intensity of internal control probes including bisulfite I, II, hybridization, extension, specificity I, II, target removal were checked and no major defects were spotted for the QC. To evaluate the presence of any batch effect, we performed multiple dimensional scaling (MDS), a dimensional reduction approach to visualize the distances (similarities) of individual cases in a dataset, using the function mdsPlot in the package “minfi”, on the 1000 most variable positions of the merged raw data. No significant batch effect was detected while the samples’ genders were well discriminated (see Figure 1). Basic quality filtering was then performed to the control-normalized and background-subtracted data exported from Illumina software GenomeStudio. Stringent data filtering was done according to recent recommendations [14] to control statistical power and reduce false discoveries. In particular, loci with detection *p*-value > 0.01 were removed along with loci having more than 20% NAs (number of detecting beads < 3) in control or case. Potential confounding factors were additionally controlled by removal of the X/Y chromosomes. Dispersion filtering measured by standard deviation (SD) and interquantile range (IQR) with cutoffs set to remove 80% of the least varying loci [14] was also performed. To achieve stringent filtering, at this stage, the intersection of the results of the 2 metrics (SD and IQR) was preserved, overall reducing the candidate loci list from ~450,000 (485,577) to 76,074.

### Differential methylation analysis

The approach uses multiple metrics and statistics to ensure that different and complementary characteristics are retained in the final ranked list.

Methylation differential values were quantified as: (i) *abs*(*mean*(*β*_*case*_) - *mean*(*β*_*control*_)) and (ii) *abs*(*median*(*β*_*case*_) - *median*(*β*_*control*_)).

Similarly, statistical tests (*p*-value < 0.05) were run with: (i) Wilcoxon rank-sum test (WRST, [15]) and (ii) Fisher’s exact test (FET, [16]) after data discretization (see Table 1 and [17]).

**Table 1:** Methylation levels’ stratification

β	0 ~ 0.2	0.2 ~ 0.5	0.5 ~ 0.8	0.8 ~ 1.0
Classification	No methylation	Low methylation	High methylation	Full methylation

Discretization ranges used to transform methylation β data for Fisher Exact test.

**Figure 2:**
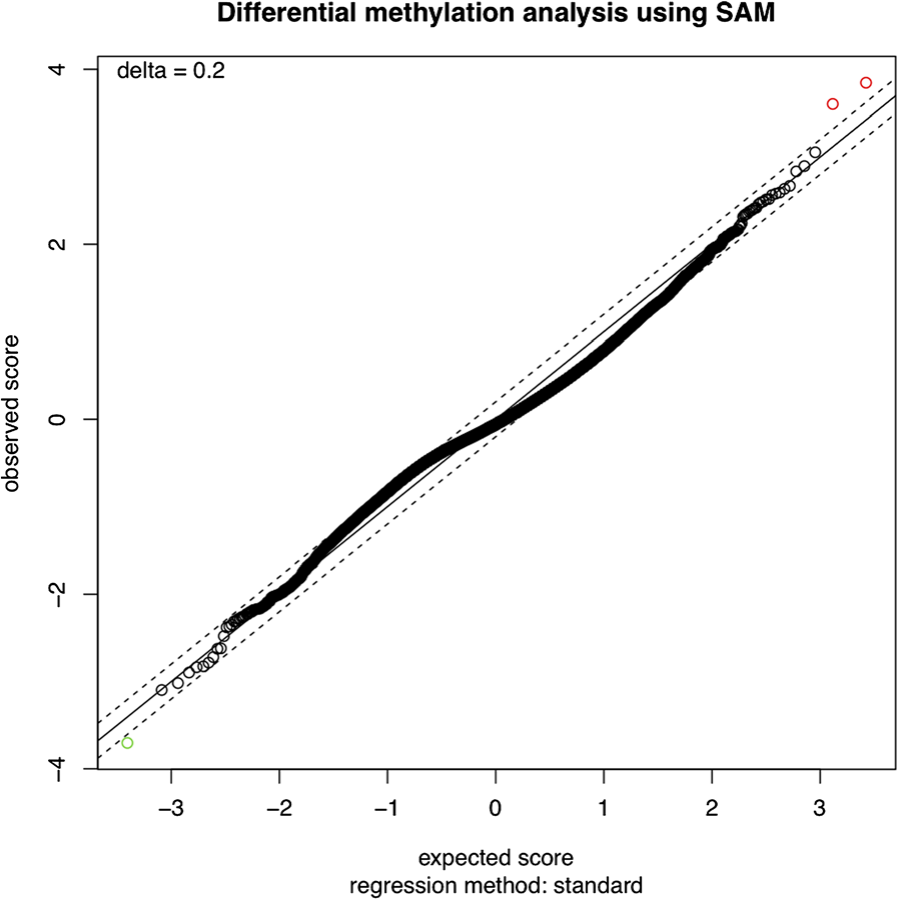
**SAM plot.** SAM plot depicting the observed *d*-statistic versus the null distribution (built by permutation) and the two lines parallel to the diagonal quantifying the deviation (effect). Ideally, points with an effect large enough, be it positive (passing the upper line on the right) or negative (passing the lower line on the left) qualify as being differential. Transcriptional SAM plots present a typical S-shape (see Additional file 7), rather than the current flat trend.

To ensure robustness of the ranking, the intersection of the statistical and differential approaches was preserved (namely: {*WRST* ∪ *FET*} ∩ {*mean* ∪ *median*}), listed in Additional file 2. Comparative analyses were also run with SAM [8] (3000 permutations and delta set to 0.2, other parameters by default) and LIMMA [9] (prior estimation of DMs set to 0.002, based on the goal of obtaining 150 candidates, others parameters by default) results are shown in Figure 2 and Table 2, respectively.

**Table 2:** Top 10 DM loci obtained with LIMMA

**Locus**	**Gene**	**Mean β**	**t-statistic**	**P-value**	**Adjusted p-value**	**B-statistic**
cg27427514	-	0.103	-5.541	6.96E-06	0.529	**2.195**
cg19938535	LRRC16A	0.668	4.980	3.15E-05	1.000	**0.808**
cg25548594	-	0.322	-4.717	6.39E-05	1.000	**0.158**
cg01821429	-	0.171	-4.697	6.75E-05	1.000	**0.107**
cg21899558	PRKAR1B	0.820	4.679	7.09E-05	1.000	**0.063**
cg01600516	ALOX12	0.690	4.376	1.60E-04	1.000	-0.688
cg26143874	-	0.815	4.252	2.23E-04	1.000	-0.991
cg15773974	-	0.629	4.167	2.81E-04	1.000	-1.201
cg01074767	C1RL; LOC283314	0.597	-4.046	3.87E-04	1.000	-1.495
cg03424554	WWP2	0.467	-3.990	4.49E-04	1.000	-1.633

Column 1 contains the locus ID; Column 2 the gene symbol(s) to which the probe is annotated; Column 3 the average methylation level (β) across all samples; Column 4 moderated t-statistic with standard errors shrunken to a common value; Column 5 nominal p-value; Column 6 multiple testing corrected p-value; column 7 Bayesian odd ratio of DM. In the last column in bold are the odd ratios greater than 0, suggesting differential methylation.

### Differential expression analysis

We collected data from 3 datasets in Gene Omnibus Express (GEO, [18]): GSE3049 [19] for the transcriptomic level, GSE19980 [20] and GSE22378 (http://www.ncbi.nlm.nih.gov/geo/query/acc.cgi?acc=GSE22378) for the post-transcriptomic level. All 3 studies use immortalized human hepatoma cell line HepG2 as HBV free model and HepG2.2.15, infected with HBV and transformed from HepG2, to mimic human chronic HBV infection.

The expression of mRNAs was monitored by CapitalBio cDNA 22 K long oligo dye-swap microarray, and compared between the two cell lines. Downloaded data were filtered by space- and intensity-dependent normalization (LOWESS), and already summarized as ratio changes for each probe set. No additional pre-processing was performed and fold-change was used with a cut-off 2 to select differentially expressed genes (DEs) in each comparison, leading to 478 DE genes (Additional file 3).

The 2 miRNA datasets were pre-processed (separately, as based on different versions of the CapitalBio mammalian miRNA array) by summarizing the expression value for each set of probes with the median (3 probes for one miRNA). Quality filtering was achieved by removing empty entries, probes for quality control (non-human) and data with more than 40% NAs. Overall 313 and 545 miRNAs remained in GSE19980 and GSE22378, respectively. After pre-processing, *t*-test, SAM and LIMMA were all applied for differential analysis in each dataset. The union, for completeness, of the results from the 2 datasets was finally retained (Additional file 4).

For DE miRNAs, targets were obtained by searching experimentally validated as well as predicted miRNA target databases, i.e. miRTarBase (release 2.5) [21] and TarBase (version 5) [22] for experimentally validated targets; TargetScan (release 6.2) [23] and microRNA.org (August 2010) [24] for predictions. Results are listed in Additional file 4 and details on the query settings can be found in Additional file 5.

## Results and discussion

Classical approaches were first tested to compute the differentially methylated (DM) loci. Although Student *t*-test [25] has been found to be applied to 450 K microarray data [26], data distribution (see Additional file 6) presents a clearly non-normal behaviour, limiting therefore the validity of the test. Similarly, while SAM [8] and LIMMA [9] do not require a rigorous normal distribution and -especially the latter- shows good performance when the sample size is small, we observed that they are not robust enough for cases showing dramatic deviation from normality, a fact also mentioned in LIMMA’s manual.

Figure 2 presents the results of SAM, where the *d-*statistic (deviation stabilized derivative of *t*-statistic, free from the normality assumption) obtained from the real (observed) data versus the null (permuted) data is plotted. Only 3 loci were identified as differential (2 hypermethylated in red, 1 hypomethylated in green), and this cannot be remedied by lowering the threshold because of the peculiar “flat” shape of this SAM curve (compared with a plot from normally distributed data, and showing the well-known SAM “S” shape plot, see Additional file 7). Indeed, although SAM exploits permutations to obtain an empirical distribution of the *d-*statistic, it is largely subject to the outliers (extreme value towards 1 or 0), which are quite frequent and indeed expected in methylomic data, and cannot be simply discarded as they are biologically valid.

LIMMA [9] computes the *B-*statistic (posterior odds statistic) by replacing the ordinary standard deviations with posterior residual standard deviations, resulting in a far more stable inference even when the sample size is small. *B-*statistic denotes the log-ratio of a locus to be differentially methylated over not being methylated, with B > 0 implying *p* > 0.5, dependent on the prior knowledge of the proportion of differential loci. In our instance, this proportion was set to 0.002, based on the future planned experimental step, which implies the validation of 150 selected loci. Table 2 shows the top 10 differential loci obtained. Although the nominal *p*-values are significant, the log-ratios (B) indicate only 5 loci with -very weak- differential signals, which confirms the caution recommended when applying LIMMA to Illumina methylation platforms [27].

Given the intrinsic difficulties in isolating statistically significant differentially methylated loci, due to the numerosity of epigenomic data (curse of dimensionality and multiple hypothesis testing issue), we chose to explore the data with two metrics to combine their diverse advantages, and to support the ranking offered by these measures with two non-parametric statistical tests.

To satisfy the biological rationale that a differential locus should present a variation in β between two phenotypes, we quantified this difference as: (i) *abs*(*mean*(*β*_*case*_) - *mean*(*β*_*control*_)) to take into account all values presented by the data and (ii) *abs*(*median*(*β*_*case*_) - *median*(*β*_*control*_)) to better deal with outliers. These values were assumed to be of relevance if above a threshold, usually set to 0.2 [14].

This ranked selection was backed by the ranking obtained from two non-parametric tests, namely Wilcoxon rank-sum test (WRST, [15]) and Fisher’s exact test (FET, [16]) after methylation status discretization [17]. Both statistical tests are free from any assumption on data distribution, yet their sensitivity and specificity vary. Wilcoxon rank-sum test is sensitive to rank orders of β values, rather than to absolute values. For this reason, we added Fisher’s exact test to the statistical framework to retain the otherwise missing information on β value.

This recommendation combined with our 150 top candidate selection lead to threshold = 0.17 (Additional file 2).

**Figure 3:**
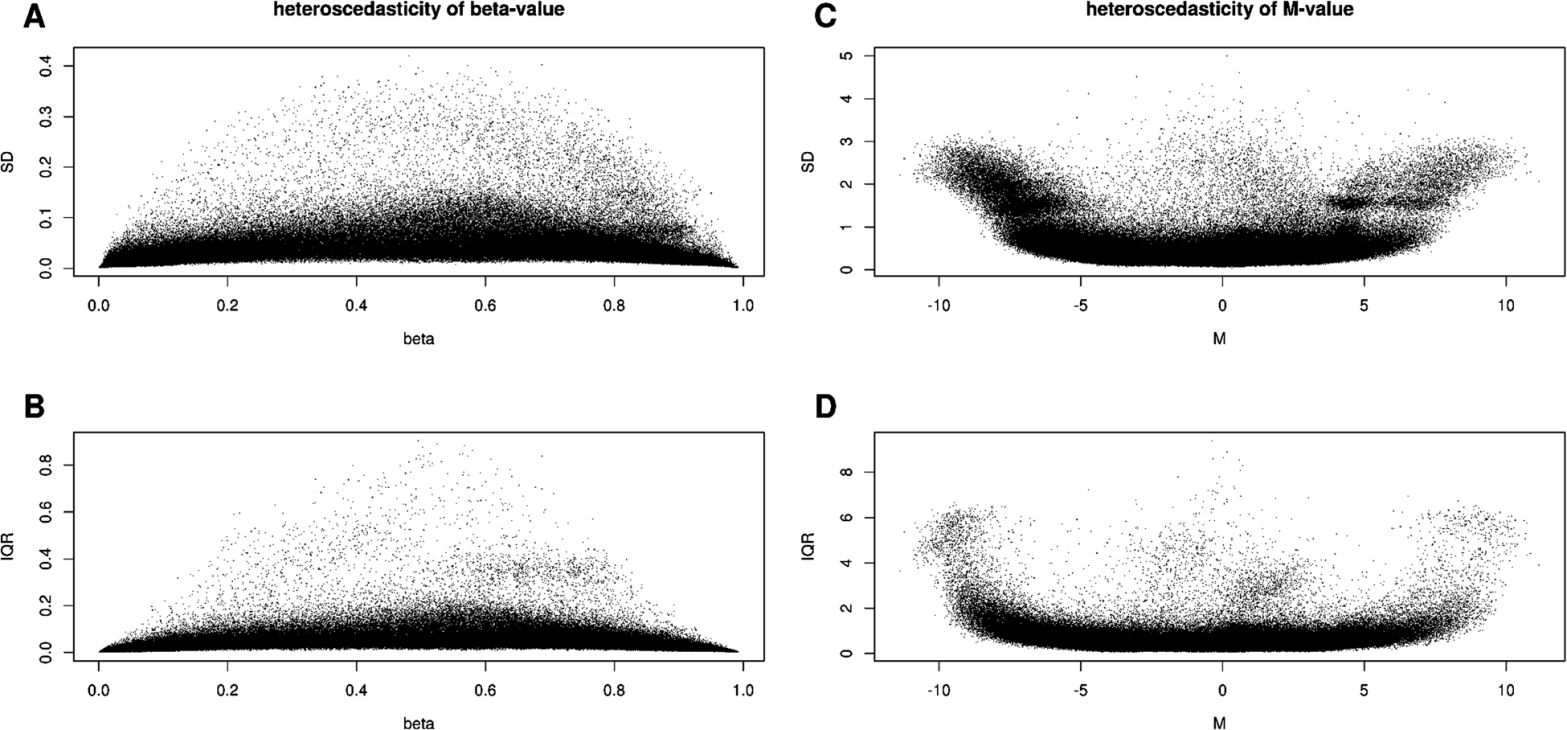
**Variance-vs-mean plot of β and M.** Panels **A**, **B** show the dependence of variance (SD in the upper panels and IQR in the lower panels) on mean values of β. Panels **C**, **D** report the same plots for M (logit transformation of β). Both show a bias of the dispersion especially when SD is used as variance indicator, either toward the middle (for β) or toward the 2 extremes (for M). Collectively these plots show that the *logit* transformation does not significantly improve heteroscedasticity.

Besides, although literature indicates the advantage of M-value (derived from the logit transformation of β: $M=\log\left(\frac{\beta }{1-\beta }\right)$), over the raw β value, since β exhibits more heteroscedasticity than M [14,28,29], we still maintained β as the metric of choice since: (i) we compared the heteroscedasticity between M and β, and, in our data, both show severe dependence of the variance on the mean and there is no clear advantage of M-value transformation as it is shown in Figure 3; (ii) to the best of our knowledge, there has been no studies as to delineate the origin of such heteroscedasticity and no rationale as to claim it is a biologically valid feature or a technical artefact, (iii) the biological meaning of β value is more intuitive.

**Figure 4:**
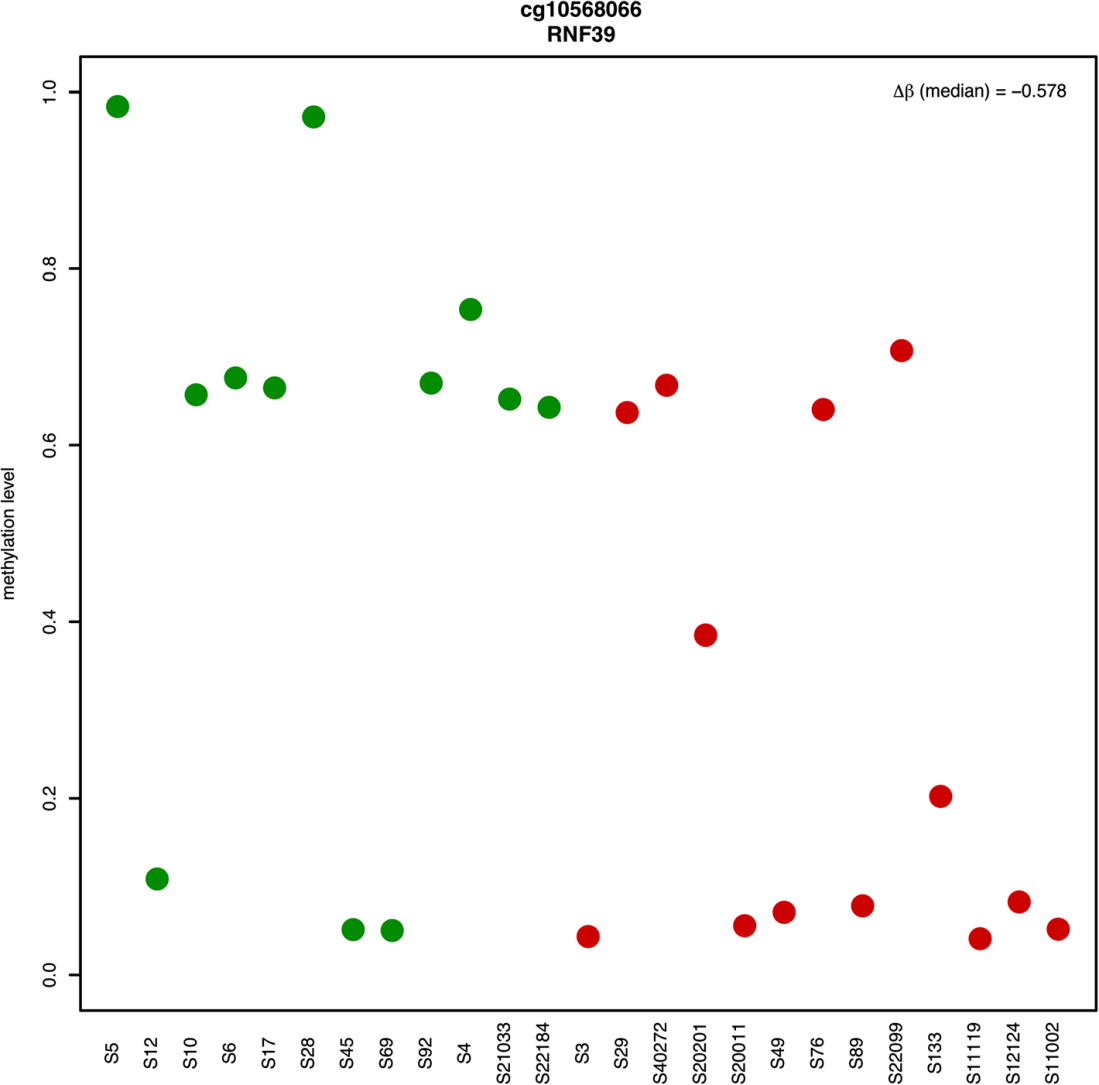
**Methylation state of cg10568066 - the most significantly differential locus annotated to RNF39.** Among the 8 differentially methylated loci annotated to RNF39 (7 hypo- and 1 hyper- methylated), cg10568066 shows the maximum difference as it is hypomethylated by 0.578.

The whole DM process led us into the identification of 146 differentially methylated loci, including several corresponding to RNF39 (the most significant one shown in Figure 4), a transcription factor in the MHC (Major Histocompatibility Complex) class I region, crucial in immune responses. This, along with the number of instances discovered, let us speculate that RNF39’s compromised methylation state may be related to poor immune responses.

To test whether altered methylation states could also be mirrored and supported in altered genes’ expression, we appended to the DM analysis a careful selection of gene expression data at both the transcriptional and post-transcriptional levels from GEO (see Methods). Due to the lack of available blood samples data we turned to hepatic cell lines, implying the assumption that the systemic effects visible in blood mirror events occurring in the disease target organ (liver), a fact that has been observed and confirmed in numerous diseases [30-32]. Differential mRNA and miRNA analyses allowed us to identify 478 DE mRNAs (Additional file 3) and 55 DE miRNAs (Additional file 4).

**Figure 5:**
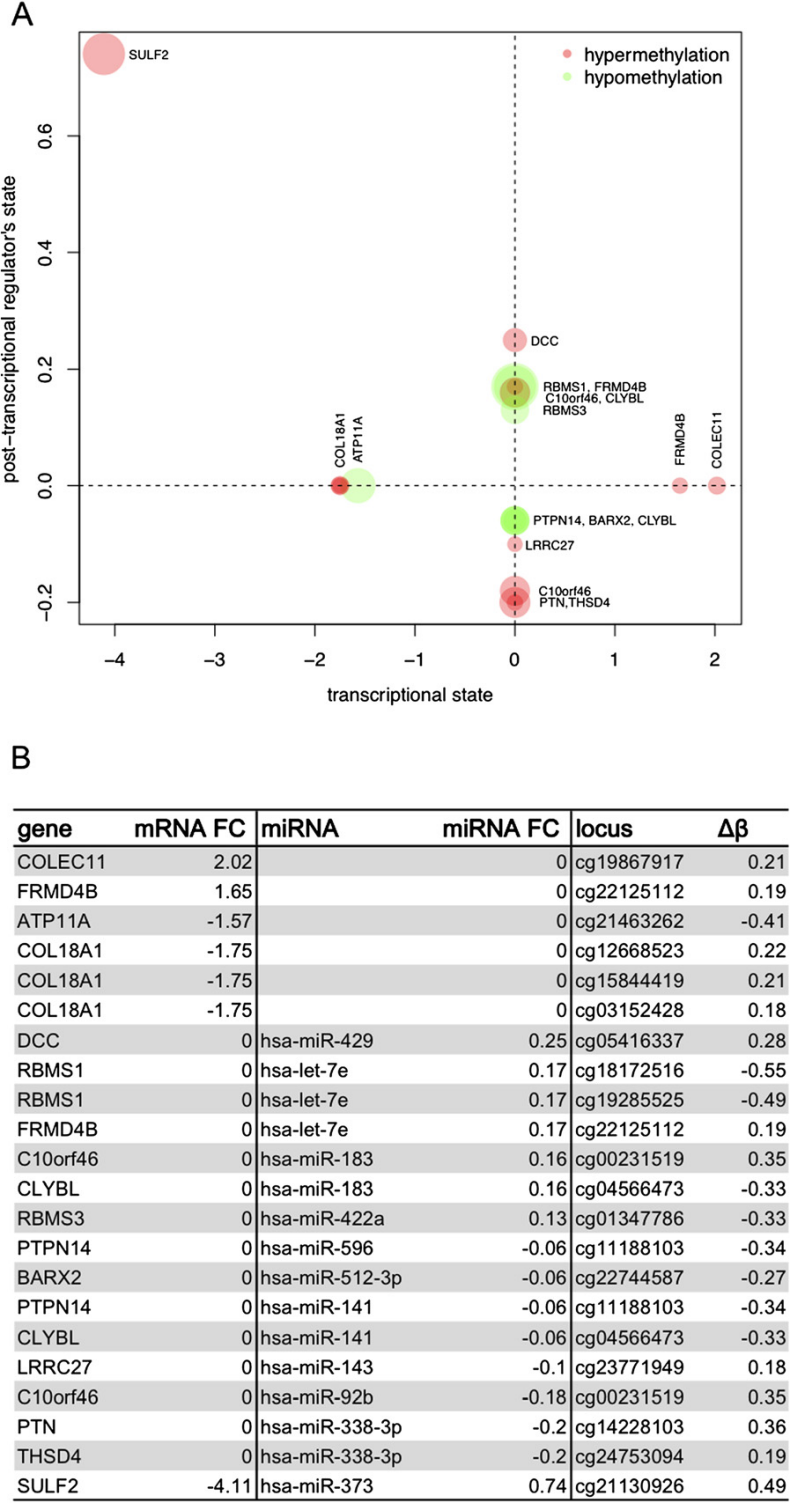
**Transcriptional and post-transcriptional regulation of DM loci genes.** In panel **A**, transcriptional (x-axis) and post-transcriptional (y-axis) differential expression (DE, log fold change) related to the genes on the differentially methylated (DM) loci (average β differences, data points’ diameters. SULF2 only falls beyond the axes, indicating contribution of all 3 variations (transcriptional, post-transcriptional, methylation) to its final state. Panel **B** quantifies further such variations.

**Figure 6:**
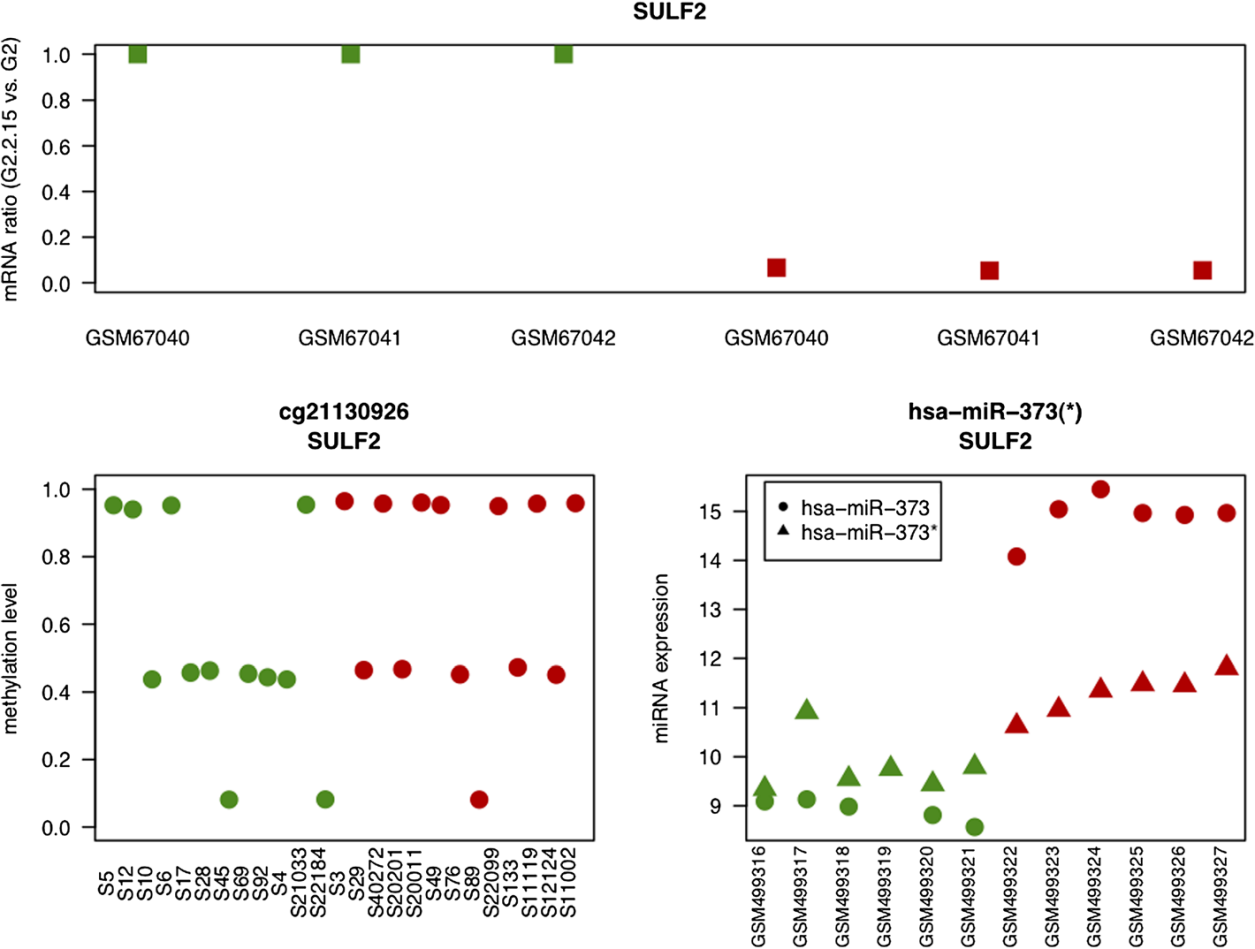
**Transcriptional and post-transcriptional regulation of SULF2.** The upper panel shows gene expression change of SULF2 (cg 21130926) in the HBV infection model (green: before infection, red: after infection). The lower panel depicts 2 possible mechanisms for the control of SULF2: transcriptional (lower left) and post-transcriptional (lower right).

The comparison between the DM and DE lists let emerge the covariation of methylomics, transcriptomics, and post-transcriptomics (Figure 5). In particular, SULF2 presents a unique situation, being the only molecule affected by variations at all 3 biochemical levels: hypermethylation and downregulation (Figure 6), along with the up-regulation of one of its controlling miRNAs (hsa-miR-373). Together, these results indicate that SULF2’s presence is likely to be extremely modest in non-responders. The gene SULF2 is known to be upregulated in 60% of primary HCCs [33], and therefore proposed as a therapeutic target [34]. Translating this information into clinical terms, it is unlikely that HCC patients who were non-responders to HBV vaccine, fall into the 60% patients that see this gene up-regulated, and hence may benefit from anti-SULF2 treatments. Therefore, based on SULF2’s screening and HBV vaccination history they could be efficiently redirected to other types of treatments.

## Conclusions

Epigenomic alterations have recently been discovered as molecular modifications with the potential to stably influence biological systems. Yet, the challenges involved in processing this type of information are numerous, including not only the biological mechanisms triggering and maintaining these modifications, but also the mathematical modelling behind these data, and from there the definition of appropriate methods of analysis. We propose here a combination of approaches to efficiently explore these data and effectively rank a selected number of epigenomic potential causes. In particular, we have been able to highlight the hypomethylation of the transcription factor RNF39 and the controlled expression of SULF2 as two interesting molecular variations related to the HBV vaccine responsiveness.

## Abbreviations

HBV: Hepatitis B virus; HCC: Hepatocellular carcinoma; DM: Differential methylation; DE: Differential expression; SAM: Significance analysis of microarrays; LIMMA: Linear models for microarray data; WRST: Wilcoxon rank-sum test; FET: Fisher’s exact test; MDS: Multiple dimensional scaling.

## Competing interests

The authors declare that they have no competing interest.

## Authors’ contributions

WY conceived the clinical study; WY and YC performed the experiments; CN and YL conceived the bioinformatics study; YL analyzed all data; YL and CN prepared the manuscript. All authors read and approved the final manuscript.

## Pre-publication history

The pre-publication history for this paper can be accessed here:

http://www.biomedcentral.com/1755-8794/7/12/prepub

## Supplementary Material

Additional file 1 **Sample labels.** Summary of clinical parameters. Click here for file

Additional file 2 Table of the DM. Click here for file

Additional file 3 Differential results for mRNA data. Click here for file

Additional file 4 **DE settings and differential results of miRNA data.** List of miRNA targets by experiments. List of miRNA targets by prediction. List of miRNA targets by both. Click here for file

Additional file 5 Parameters used in predicting miRNA targets. Click here for file

Additional file 6 Bean plot showing β distribution. Click here for file

Additional file 7 SAM plot (and its code), which exemplifies application to normally distributed data. Click here for file
