# Supplementary material for: Exploring the molecular causes of hepatitis B virus vaccination response: an approach with epigenomic and transcriptomic data
Source: BMC Med Genomics. 2014 Mar 11;7:12. doi: 10.1186/1755-8794-7-12 (PMC4008305; doi:10.1186/1755-8794-7-12)
Supplement: Additional file 5 — Parameters used in predicting miRNA targets. [file 1755-8794-7-12-S5.docx]

Parameters for target prediction algorithms:

1. *TargetScan*
   1. for conserved miRNA: context score percentile < 20% and context score < -0.2;
   2. for noconserved miRNA: context score percentile < 5% and context score < -0.05; then both conserved and nonconserved targets were combined

finally a) and b) are merged.

1. *microRNA.org*

miRanda conserved and "good score" miRNA with mirSVR score percentile < 10%, miSVR score < -0.6, energy percentile < 10%, and energy < -19
