# Supplementary material for: Exploring the molecular causes of hepatitis B virus vaccination response: an approach with epigenomic and transcriptomic data
Source: BMC Med Genomics. 2014 Mar 11;7:12. doi: 10.1186/1755-8794-7-12 (PMC4008305; doi:10.1186/1755-8794-7-12)
Supplement: Additional file 6 — Bean plot showing β distribution. [file 1755-8794-7-12-S6.docx]

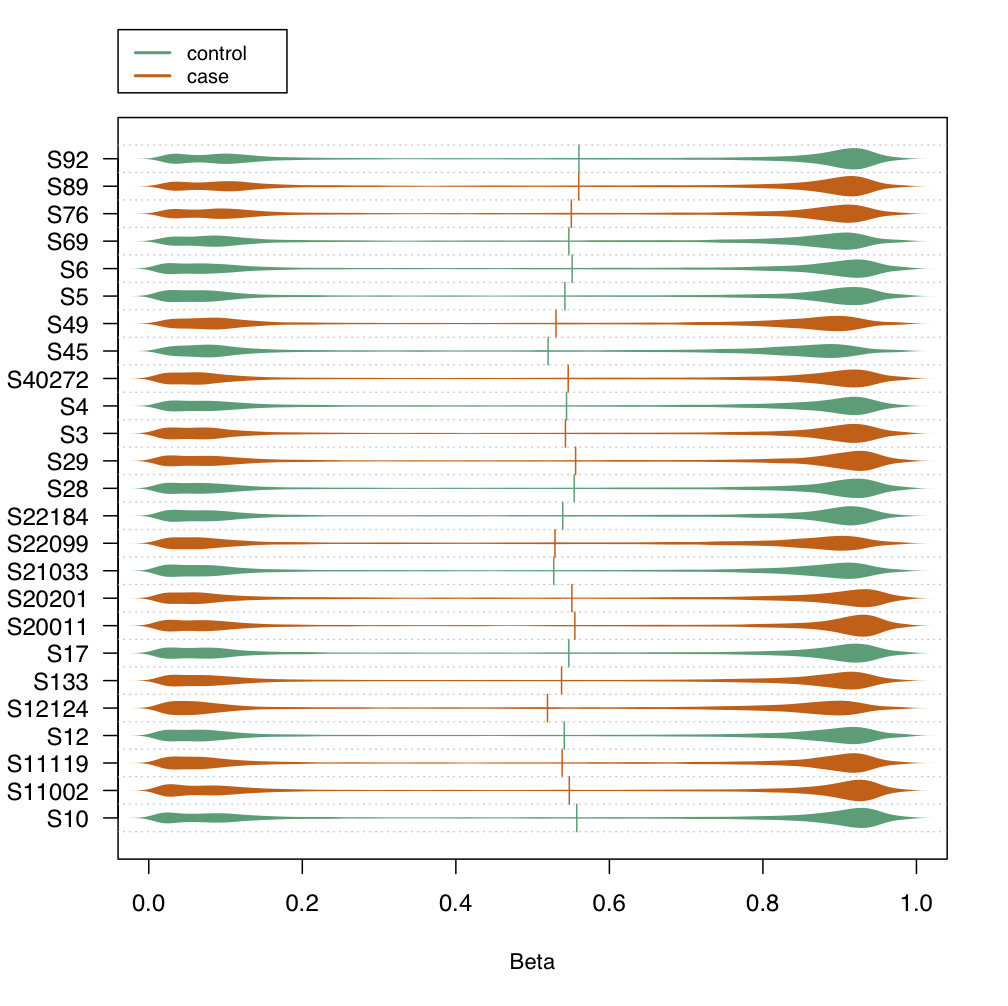


Figure. Bean plot showing the beta distribution. Data distribution as density shape combined with one-dimensional scatter plot for each measurement, with the middle vertical bar indicating the overall average, more suitable for QC than density plot. As the figure shows, in each sample there are two bulbs at the low and high end of the β region, building a bimodal distribution, which reflects the biological fact that most of the CpG loci are either hypomethylated or hypermethylated. No obvious defects were identified.
