# Supplementary material for: Exploring the molecular causes of hepatitis B virus vaccination response: an approach with epigenomic and transcriptomic data
Source: BMC Med Genomics. 2014 Mar 11;7:12. doi: 10.1186/1755-8794-7-12 (PMC4008305; doi:10.1186/1755-8794-7-12)
Supplement: Additional file 7 — SAM plot (and its code), which exemplifies application to normally distributed data. [file 1755-8794-7-12-S7.docx]

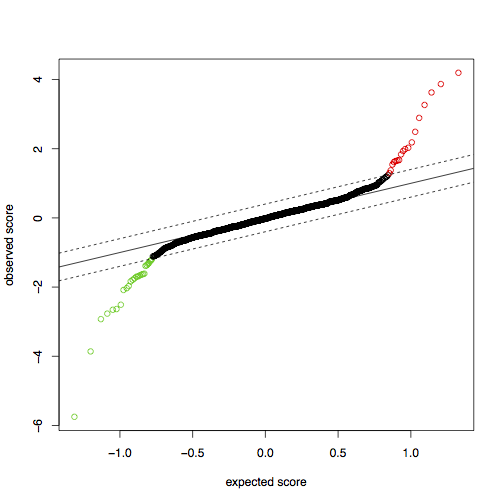


Figure. SAM application to normal distributed data. An example of SAM plot taken from package *samr* where 10% of features are set differential to an overall normal distributed data. Notice the trend of points distinctive from ours (see Figure 2 in the main manuscript). The code to generate the plot:

######### two class unpaired comparison

# y must take values 1,2

set.seed(100)

x <- matrix(rnorm(1000 * 20), ncol = 20) # data based on normal distribution

dd <- sample(1:1000, size = 100)

u <- matrix(2 * rnorm(100), ncol = 10, nrow = 100)

x[dd, 11:20] <- x[dd, 11:20] + u

y <- c(rep(1, 10), rep(2, 10))

data = list(x = x, y = y, geneid = as.character(1:nrow(x)), genenames = paste("g", as.character(1:nrow(x)), sep = ""), logged2 = TRUE)

samr.obj <- samr(data, resp.type = "Two class unpaired", nperms = 100)

delta = .4

samr.plot(samr.obj,delta)
